# Efficacy of Empagliflozin on Liver Enzymes, Lipid Profile and BMI in Patients With Metabolic Dysfunction–Associated Steatotic Liver Disease (MASLD): A Systematic Review and Meta‐Analysis

**DOI:** 10.1002/edm2.70294

**Published:** 2026-07-26

**Authors:** Mehdi Karimi, Ali Tahmasebi, Sepehr Ramezanipour, Fatemeh Naseri Rad, Fereshteh Valizadeh, Pegah Abedi, Meisam Gholami, Narges Sobhan Ardekani, Manijeh Ebrahimzadeh Pirshahid

**Affiliations:** ^1^ Faculty of Medicine Bogomolets National Medical University (NMU) Kyiv Ukraine; ^2^ Student Research Committee Tabriz University of Medical Sciences Tabriz Iran; ^3^ Student Research Committee Fasa University of Medical Sciences Fasa Iran; ^4^ Student Research Committee, School of Medicine Iran University of Medical Sciences Tehran Iran; ^5^ School of Medicine Shahid Beheshti University of Medical Sciences (SBUMS) Tehran Iran; ^6^ Faculty of Medicine Isfahan University of Medical Sciences Isfahan Iran; ^7^ Faculty of Medicine Islamic Azad University, Tehran Medical Science Branch Tehran Iran; ^8^ Student Research Committee, Health Research Institute Babol University of Medical Sciences Babol Iran

**Keywords:** empagliflozin, fatty liver disease, hepatic steatosis, lipid profile, liver enzymes, meta‐analysis

## Abstract

**Background:**

Metabolic dysfunction‐associated steatotic liver disease (MASLD) is increasingly prevalent and associated with obesity and dyslipidemia, yet effective pharmacological interventions remain limited. Empagliflozin has shown potential benefits in MASLD, although evidence remains limited. This systematic review and meta‐analysis assesses the efficacy of empagliflozin in patients with MASLD, both diabetic and non‐diabetic.

**Methods:**

An advanced database search was conducted up to June 1st, 2026, to identify eligible randomized controlled trials (RCTs). The relevant data were extracted from selected studies. Pooled effect sizes were calculated as weighted mean differences (WMDs) with 95% confidence intervals (CIs) using a random‐effects model.

**Results:**

Seven RCTs comprising 591 participants were included. Empagliflozin significantly improved liver enzyme profiles. Specifically, it reduced alanine aminotransferase (ALT) (WMD: −12.24 IU/L; *p* = 0.037), aspartate aminotransferase (AST) (WMD: −9.51 IU/L; *p* = 0.048), and gamma‐glutamyl transferase (GGT) (WMD: −14.92 IU/L; *p* = 0.023). Reductions in alkaline phosphatase (ALP) were observed but did not reach statistical significance (WMD: −26.28 IU/L; *p* = 0.292). Although substantial heterogeneity was observed for several liver enzyme outcomes. Empagliflozin had no significant effect on lipid parameters, including triglycerides (TG), total cholesterol (TC), low‐density and high‐density lipoprotein cholesterols (LDL‐C, HDL‐C). Empagliflozin also produced a statistically significant but modest reduction in body mass index (BMI) (WMD: −0.71 kg/m^2^; *p* < 0.001). Empagliflozin also produced a statistically significant but clinically modest reduction in BMI (WMD: −0.71 kg/m^2^; 95% CI: −1.00 to −0.43; *p* < 0.001).

**Conclusion:**

Empagliflozin appears to improve liver enzymes and modestly reduce BMI in patients with MASLD, while having a neutral effect on lipid parameters. These findings may confer a statistically significant but clinically modest benefit, particularly in patients with coexisting metabolic risk factors. However, the evidence is limited by small sample sizes, short follow‐up periods, and potential publication bias; therefore, the conclusions should be considered preliminary and hypothesis‐generating.

## Graphical abstract

This random‐effects meta‐analysis of 7 randomized controlled trials involving 591 patients with MASLD, with or without type 2 diabetes, found that empagliflozin significantly improved liver enzymes, reducing ALT by 12.24 IU/L, AST by 9.51 IU/L, and GGT by 14.92 IU/L, while modestly reducing BMI by 0.71 kg/m². No significant effects were observed on lipid parameters, including triglycerides, total cholesterol, LDL‐C, or HDL‐C. Although the findings suggest a promising therapeutic role for empagliflozin in MASLD, the evidence remains preliminary due to the small number of trials, short follow‐up durations, substantial heterogeneity, and possible publication bias, underscoring the need for larger, long‐term randomized controlled trials.

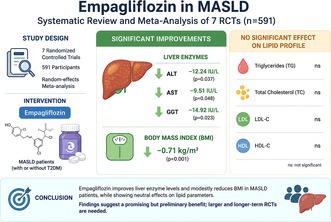

**Abbreviations**
ALP: alkaline phosphatase
ALT: alanine aminotransferase
AST: aspartate aminotransferase
BMI: body mass index
CI: confidence intervals
GGT: gamma‐glutamyl transferase
HDL‐C: high‐density lipoprotein cholesterol
LDL‐C: low‐density lipoprotein cholesterol
MASLD: metabolic dysfunction‐associated steatotic liver disease
NAFLD: non‐alcoholic fatty liver disease
NASH: non‐alcoholic steatohepatitis
RCT: randomized controlled trials
SD: standard deviation
SGLT‐2: sodium–glucose cotransporter‐2
T2DM: type 2 diabetes mellitus
TC: total cholesterol
TG: triglycerides
WMD: weighted mean difference

## 1 Introduction

Metabolic dysfunction–associated steatotic liver disease (MASLD) is a novel terminology that has replaced the former term non‐alcoholic fatty liver disease (NAFLD) [1]. A more advanced form, non‐alcoholic steatohepatitis (NASH), is characterized by liver inflammation and hepatocyte injury and may progress to fibrosis, cirrhosis and other liver‐related complications [2]. Unlike previous definitions, MASLD may coexist with excessive alcohol intake, concurrent liver diseases and other secondary causes of steatosis [3]. Diagnosis is based on evidence of hepatic steatosis, assessed by histology or imaging, in the presence of one or more cardiometabolic risk factors [4]. MASLD is now recognized as a major public health concern, with an estimated prevalence of more than one‐third of the adult population worldwide [5]. Its pathogenesis is closely linked to overweight and obesity, insulin resistance, alterations in gut microbiota and genetic susceptibility, with the rising prevalence of obesity and type 2 diabetes mellitus (T2DM) serving as key drivers of disease burden. Consequently, targeting underlying metabolic abnormalities represents a central therapeutic strategy [5, 6]. In this context, recent studies have suggested that certain pharmacological agents, such as orlistat [7, 8] or N‐acetylcysteine [9], and dietary supplementation may lead to improvements in metabolic parameters and liver‐related outcomes in patients with MASLD [10, 11].

Empagliflozin, a sodium–glucose cotransporter‐2 (SGLT‐2) inhibitor, serves as an effective glucose‐lowering agent for adults with T2DM. It acts through an insulin‐independent mechanism by reducing the renal threshold for glucose reabsorption, thereby promoting glycosuria and consequently lowering plasma glucose [12]. Furthermore, empagliflozin suggests favourable effects on non‐glycemic outcomes, such as a reduction in body weight, visceral adiposity and blood pressure [13, 14]. Additionally, it influences the metabolism of ketone bodies, lipids, and fatty acids. These benefits suggest potential for improving liver parameters, although evidence from clinical practices is still limited [15].

Emerging evidence suggests that SGLT‐2 inhibitors, particularly empagliflozin, may confer beneficial effects in patients with MASLD [16, 17]. A recent clinical trial suggested that empagliflozin significantly reduced hepatic steatosis and lowered liver enzyme levels compared with control therapy [18]. However, the existing evidence remains inconclusive. Although two meta‐analyses have been published, their findings are limited by the small number of included studies and warrant updating [19, 20]. Zhang et al. [19] pooled four randomized controlled trials (RCTs) and reported improvements in body composition, insulin resistance, liver fibrosis and hepatic enzymes in patients with NAFLD, supporting empagliflozin as a potential therapeutic option. In contrast, the meta‐analysis by Tang et al. [20], which included only three studies, found no significant effects on hepatic outcomes, lipid profile or liver stiffness. These conflicting results highlight the need for a more comprehensive, up‐to‐date synthesis to clarify the therapeutic efficacy of empagliflozin.

Therefore, this systematic review and meta‐analysis of RCTs was conducted to comprehensively update and evaluate the effects of empagliflozin on liver enzymes, lipid profile and body mass in patients with MASLD with and without diabetes mellitus, to synthesize the available clinical evidence and provide more robust and conclusive insights into its potential role in MASLD management.

## 2 Methods

### 2.1 Study Protocol and Design

This systematic review and meta‐analysis was conducted in accordance with the Preferred Reporting Items for Systematic Reviews and Meta‐Analyses (PRISMA 2020) guidelines [21]. The research question and eligibility criteria were structured using the *PICOS* framework (Population, Intervention, Comparison, Outcomes and Study Design) [22] as follows:

- P (population): Adult patients diagnosed with MASLD/NAFLD or NASH, with or without T2DM
- I (intervention): Oral administration of empagliflozin
- C (comparison): Placebo, standard care or no treatment
- O (outcomes): Changes in Liver Enzymes, including aspartate aminotransferase (AST), alanine aminotransferase (ALT), gamma‐glutamyl transferase (GGT), serum lipid profile including triglycerides (TG), total cholesterol (TC), low‐ and high‐density lipoprotein cholesterol (LDL and HDL) and body mass index (BMI)
- S (study design): RCT

### 2.2 Search Strategy

A comprehensive systematic search was conducted across PubMed, Scopus and Web of Science to identify relevant RCTs published from inception until June 1st, 2026. The strategy combined MeSH (Medical Subject Headings) terms with non‐MeSH terms related to empagliflozin and metabolic outcomes, including serum liver enzymes, lipid profile and BMI, in patients with MASLD with and without diabetes mellitus. Keywords were searched in titles and abstracts, and Boolean operators (AND/OR) were applied to enhance sensitivity and capture all eligible studies. The keywords included: (‘Empagliflozin’ OR ‘Jardiance’) AND (‘Fatty liver’ OR ‘fatty liver disease’ OR ‘liver steatosis’ OR ‘hepatic steatosis’ OR ‘steatohepatitis’ OR ‘steatotic liver’ OR ‘Non‐alcoholic fatty liver disease’ OR ‘NAFLD’ OR ‘Nonalcoholic steatohepatitis’ OR ‘NASH’ OR ‘metabolic dysfunction‐associated steatotic liver disease’ OR ‘MASLD’). No restrictions were placed on language or date of publication. To ensure completeness, additional searches were conducted in Google Scholar and in the reference lists of included studies and relevant reviews.

### 2.3 Eligibility Criteria

The eligibility criteria were defined according to the PICO framework. Studies were included if they met all of the following criteria: (1) RCTs published as full‐text articles in scientific journals; (2) adult participants (≥ 18 years) diagnosed with MASLD, NAFLD or NASH according to the diagnostic criteria used in the original studies. Because most eligible trials predated the MASLD consensus terminology, studies enrolling patients with NAFLD or NASH were considered eligible when their reported clinical characteristics were consistent with the contemporary MASLD definition. (3) intervention consisting of empagliflozin administered at any approved dose and duration, either as monotherapy or in combination with standard treatment, compared with placebo, standard care or no intervention; (4) reporting baseline and post‐intervention data for at least one relevant outcome, enabling calculation of mean differences and corresponding measures of variance; and (5) studies conducted in clinical settings involving human participants.

Studies were excluded if they (1) were randomized trials without a control group; (2) did not include patients with a confirmed diagnosis of MASLD/NAFLD or NASH; (3) evaluated interventions other than empagliflozin; (4) failed to report relevant outcomes; (5) were non‐randomized studies, animal or in vitro experiments, case reports, case series, case–control studies, cohort studies, conference abstracts, letters, reviews or meta‐analyses; (6) lacked accessible full texts or adequate baseline and endpoint data; or (7) did not provide sufficiently detailed statistical analyses of outcome changes, thereby limiting quantitative synthesis.

### 2.4 Study Selection Process

Following advanced research on databases, all identified records were imported into *EndNote* for management and de‐duplication. Screening was performed independently by two reviewers (P.A. and M.GH.), with disagreements resolved through discussion or, if necessary, consultation with a third reviewer (M.K.). The screening process was carried out in two stages. First, titles and abstracts were reviewed to exclude studies that were clearly irrelevant. Second, the full texts of potentially eligible articles were assessed against the predefined inclusion and exclusion criteria. Any discrepancies at either stage were resolved by consensus or referral to the third reviewer.

### 2.5 Data Extraction

Data from the included RCTs were systematically extracted using a standardized data extraction form. The information collected encompassed study characteristics (author, year, country, study design and sample size), patient characteristics (age, sex, BMI and health status), intervention characteristics (empagliflozin dosage and duration), and comparator characteristics (placebo, standard treatment or control). Clinical outcomes of interest were extracted, including changes in liver enzymes (AST, ALT, ALP and GGT), lipid profile parameters (TG, TC, LDL‐C and HDL‐C), and BMI at both baseline and study endpoint. For parallel and crossover trial designs, data were reported as means ± standard deviations (SD). When pre‐ and post‐intervention values were not directly reported, mean differences were calculated by subtracting baseline values from post‐intervention values. All extracted data were organized in an Excel spreadsheet to facilitate subsequent statistical analysis. To minimize bias, data extraction was conducted independently by two reviewers (M.K. and A.T.), with any discrepancies resolved through discussion and consultation.

### 2.6 Quality Assessment

The methodological quality of the included studies was assessed across five domains using the Cochrane Risk of Bias 2.0 tool (RoB‐2) [23]. Bias arising from the randomization process (D1), bias due to deviations from intended interventions (D2), bias due to missing outcome data (D3), bias in measurement of outcomes (D4) and bias in selection of reported results (D5). Each domain was rated as ‘low risk’, ‘high risk’ or ‘some concerns’ when reporting was insufficient to allow a definitive judgement. The assessments were performed independently by two reviewers (M.K. and A.T.), and any discrepancies were resolved through discussion. An overall risk‐of‐bias judgement was assigned to each study based on the combined evaluation of all domains.

### 2.7 Statistical Analysis

All statistical analyses were performed to quantitatively synthesize the intervention's effects on the outcomes of interest. For each included RCT, mean changes from baseline to post‐intervention were extracted or calculated for continuous outcomes. Pooled effect estimates were expressed as weighted mean differences (WMDs) with corresponding 95% confidence intervals (CIs). Owing to the anticipated clinical and methodological heterogeneity across studies, including differences in participant characteristics, intervention duration and baseline disease severity, random‐effects models were applied throughout using the restricted maximum likelihood (REML) estimator to account for both within‐ and between‐study variability. When change‐score standard deviations were unavailable, they were imputed using the Follmann method assuming a within‐group correlation coefficient (*r*) of 0.8. This value has been widely adopted in meta‐analyses of continuous outcomes when individual participant data are unavailable and provides a reasonable approximation while minimizing unnecessary exclusion of eligible studies.

For studies reporting continuous data as medians and interquartile ranges (IQRs), values were converted to means and SDs using the method proposed by Wan et al. [24]. This approach was required for only two RCTs: Cheung et al. [17] and Taha et al. [25]. Although these conversions may introduce some additional imprecision, particularly in studies with small sample sizes, their limited use is unlikely to have materially influenced the overall findings.

Between‐study heterogeneity was evaluated using Cochran's *Q* test (*p* < 0.10 indicating significant heterogeneity) and quantified using the *I* ^2^ statistic, with values of 25%, 50% and 75% interpreted as low, moderate, and high heterogeneity, respectively. Potential publication bias was assessed visually by inspecting the funnel plot and statistically using Begg's rank correlation and Egger's regression tests; a *p*‐value < 0.05 indicated asymmetry suggestive of small‐study effects. In cases of detected bias, the trim‐and‐fill method was employed to estimate adjusted effect sizes. To assess the robustness of pooled outcomes, leave‐one‐out sensitivity analyses were performed by sequentially excluding each study and recalculating the overall WMDs to determine the influence of each trial on the summary estimate. Prespecified subgroup analyses were conducted according to diabetes status (T2DM vs. non‐diabetes), BMI category (overweight: BMI 25–30 kg/m^2^; obesity: BMI ≥ 30 kg/m^2^), and baseline biochemical status using clinically relevant cut‐offs (ALT and AST: 40 IU/L; TG: 200 mg/dL; LDL‐C: 100 mg/dL; HDL‐C: 50 mg/dL). All statistical procedures were performed in R (version 4.5.1; R Foundation for Statistical Computing, Vienna, Austria) using the meta and metafor packages, with two‐sided testing and a significance threshold of *p* < 0.05.

### 2.8 Certainty Assessment

The certainty of evidence for the primary outcomes was evaluated using the GRADE (Grading of Recommendations Assessment, Development and Evaluation) framework, which considers factors such as risk of bias, study inconsistency, indirectness of evidence, imprecision of estimates and potential publication bias to classify evidence as high, moderate, low or very low [26]. Two independent reviewers conducted the assessments, with any disagreements resolved through discussion or consultation with a third reviewer. The resulting GRADE ratings informed confidence in the meta‐analytic findings and guided interpretation of the results.

## 3 Results

### 3.1 Study Selection

**FIGURE 1:**
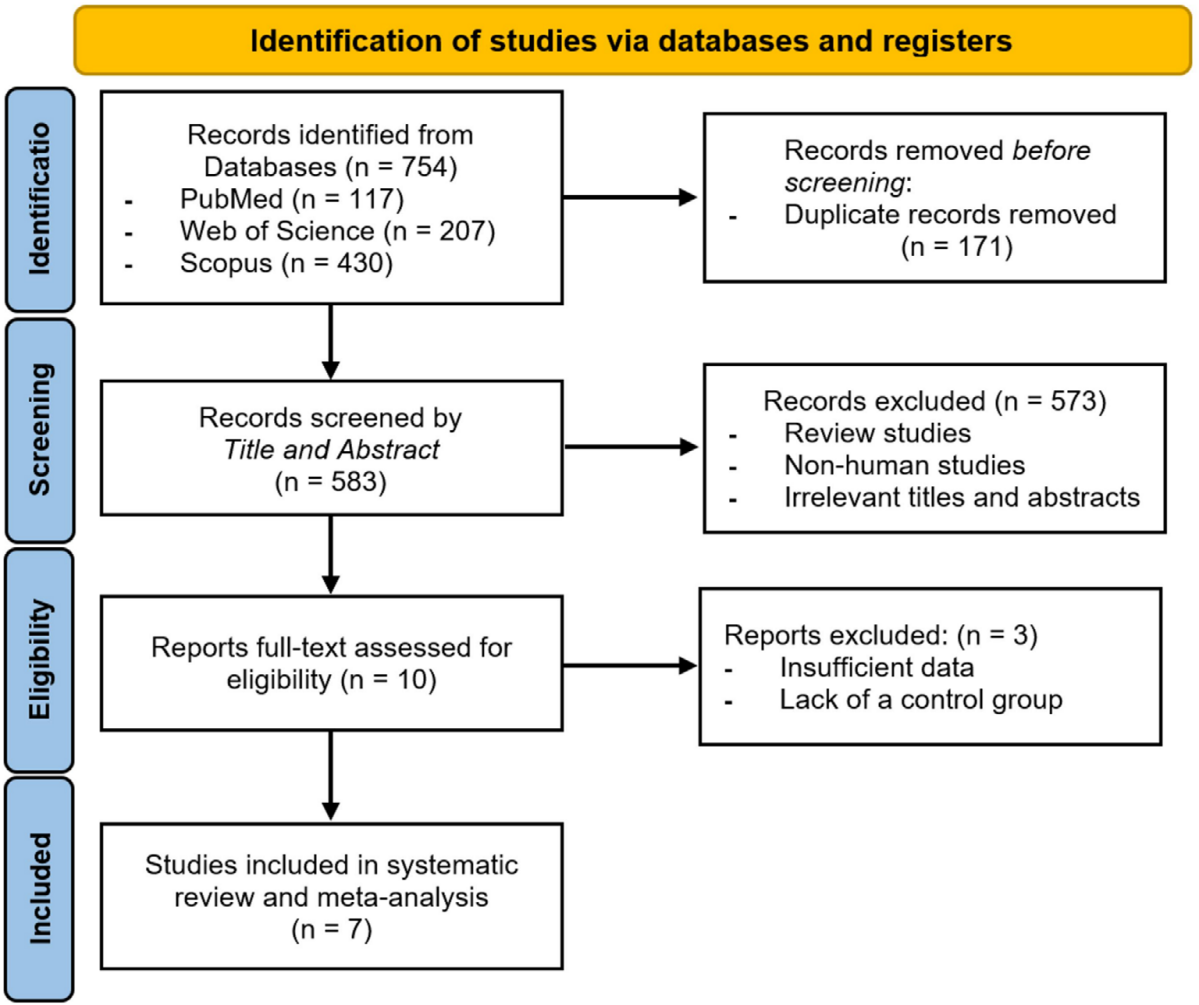
PRISMA flow chart of the study selection process for inclusion studies in the systematic review.

The literature search yielded 754 records across three databases: PubMed (*n* = 117), ISI Web of Science (*n* = 207) and Scopus (*n* = 430). After removing 171 duplicates, 583 unique records remained for title and abstract screening. During this stage, 573 articles were excluded for being unrelated to the research objectives or failing to meet the eligibility criteria. The remaining 10 studies were then assessed in detail. Following this review, three studies were excluded due to insufficient or irrelevant data, the absence of a control group or the lack of combined supplementation. Ultimately, seven RCTs [16, 17, 18, 25, 27, 28, 29] fulfilled all inclusion criteria and were included in the final systematic review (Figure 1).

### 3.2 Study Characteristics

The basic characteristics of the included trials are summarized in Table 1 [16, 17, 18, 25, 27, 28, 29]. Seven RCTs comprising 591 participants were included in the present systematic review and meta‐analysis. The studies were published between 2018 and 2025 and were conducted across four countries: Iran (*n* = 4), India (*n* = 1), China (*n* = 1), and Egypt (*n* = 1). All included studies employed a parallel‐group randomized design, and three were additionally conducted as double‐blind placebo‐controlled trials. Sample sizes ranged from 42 to 119 participants, with intervention durations varying from 20 to 24 weeks. Participants included adults with MASLD, previously classified as NAFLD or NASH, with or without concomitant T2DM. Four studies enrolled only patients with MASLD and T2DM, whereas two included non‐diabetic patients with MASLD. The diagnosis of MASLD was established using various validated imaging modalities, including magnetic resonance imaging (MRI) with proton density fat fraction (PDFF), transient hepatic elastography with controlled attenuation parameter (CAP), ultrasound or combinations thereof. None of the included RCTs used paired liver biopsy, which remains the reference standard for assessing disease activity and fibrosis. The mean age of participants ranged from approximately 44 to 56 years, while baseline BMI ranged from 26.5 to 32.2 kg/m^2^. Across all studies, the intervention consisted of empagliflozin administered at a dose of 10 mg/day. Comparator groups received placebo, standard treatment, lifestyle modification or routine care.

**TABLE 1:** Basic characteristics of included studies in the systematic review and meta‐analysis.

Study	Country	Study design	Health status	Diagnostic methods	Sex (M/F)	Sample size (IG + CG)	Trial duration (week)	Age		BMI		Intervention	Control
								IG	CG	IG	CG		
Kuchay et al. [27]	India	Parallel, RCT, PC	NAFLD with T2DM	MRI‐PDFF (6%)	NR	42 (22 + 20)	20	> 20	> 20	30.0 ± 3.8	29.4 ± 3.1	Empagliflozin (10 mg/day)	Standard Treatment
Taheri et al. [28]	Iran	Parallel, RCT, PC, DB	NAFLD	Ultrasound or MRI‐PDFF	M: 50 F: 40	90 (43 + 47)	24	43.8 ± 9.7	44.1 ± 9.3	30.5 ± 2.3	30.7 ± 3.5	Empagliflozin (10 mg/day)	Placebo
Chehrehgosha et al. [29]	Iran	Parallel, RCT, PC, DB	NAFLD with T2DM	Transient hepatic elastography (CAP ≥ 238 dB/m)	M: 29 F: 48	77 (35 + 37)	24	51.8 ± 7.8	50.5 ± 8.4	30.9 ± 3.3	30.2 ± 4.4	Empagliflozin (10 mg/day)	Placebo
Cheung et al. [17]	China	Parallel, RCT, PC, DB	NAFLD without DM	Transient hepatic elastography (CAP ≥ 268 dB/m)	M: 54, F: 41	98 (49 + 49)	24	56.4 ± 10.22	54.4 ± 10.29	27.4 ± 3.85	27.1 ± 2.88	Empagliflozin (10 mg/day)	Placebo
Nasiri Mehr et al. [16]	Iran	Parallel, RCT, PC	NASH with T2DM	(Non‐reported)	M: 56, F: 54	110 (55 + 55)	24	50 ± 14 (22–78)	50 ± 14 (22–78)	26.53 ± 3.03	26.53 ± 3.03	Empagliflozin (10 mg/day)	Placebo
Shojaei et al. [18]	Iran	Parallel, RCT, PC	NAFLD with T2DM	Ultrasound and MRI	M: 70 F: 49	119 (69 + 50)	24	46.32 (8.11)	52.56 (10.26)	32.18 ± 4.24	31.13 ± 6.05	Empagliflozin (10 mg/day)	Control
Taha et al. [25]	Egypt	Parallel, RCT, PC	NAFLD without DM	Transient hepatic elastography (CAP ≥ 268 dB/m)	M: 37, F: 18	55 (33 + 22)	24	42 ± 22.95 (35–52)	42 ± 22.95 (35–52)	30.8 (29.4–31.8)	30.9 (30.5–31.4)	Empagliflozin (10 mg/day)	Lifestyle modification

Abbreviations: CAP, controlled attenuation parameter; CG, control group; DB, double‐blind; F, female; IG, intervention group; M, male; MRI, magnetic resonance imaging; NAFLD, non‐alcoholic fatty liver disease; PC, placebo‐control; PDFF, proton density fat fraction; RCT, randomized clinical trial; T2DM, type 2 diabetes mellitus.

Although most included trials originally used the historical NAFLD or NASH terminology, all enrolled participants met metabolic criteria consistent with the current MASLD definition under contemporary international nomenclature.

### 3.3 Risk of Bias Assessment

**FIGURE 2:**
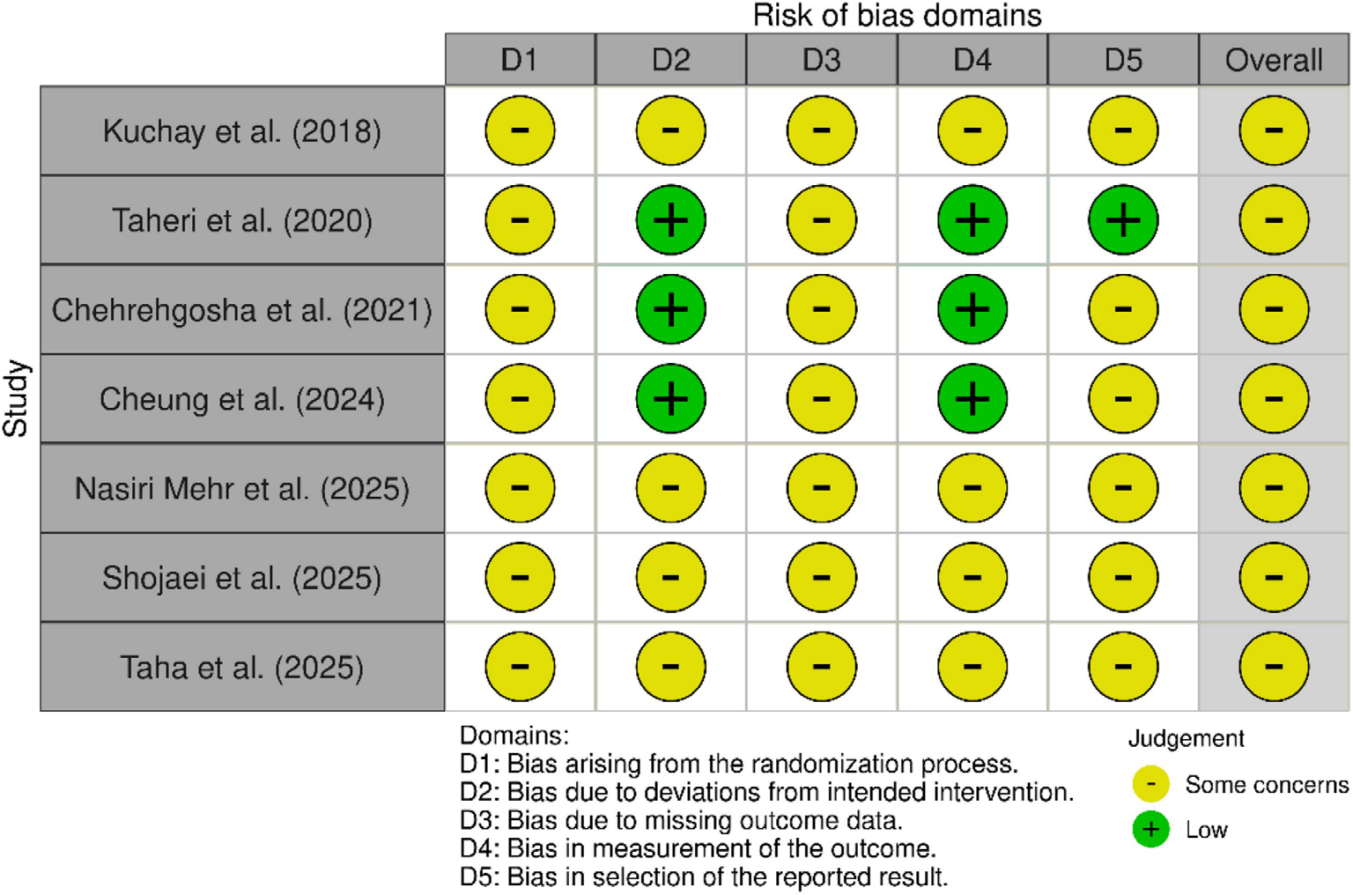
Quality assessment of studies according to Cochrane risk‐of‐bias 2 (RoB‐2).

The quality assessment of the seven studies included in this meta‐analysis revealed that most were rated as having ‘some concerns’ across multiple domains. Specifically, all studies raised concerns about bias arising from the randomization process (D1). Domains related to deviations from intended interventions (D2) and measurement of outcomes (D4) were frequently rated as low risk in three studies, while the remaining studies had some concerns. Bias due to missing outcome data (D3) and to the selection of reported results (D5) raised some concerns. Overall, no study was judged to be at high risk of bias, and the general trend indicates that insufficient reporting limited the certainty of risk assessments in several domains (Figure 2).

### 3.4 Meta‐Analysis Results

#### 3.4.1 Effect of Empagliflozin on Liver Enzymes

**FIGURE 3:**
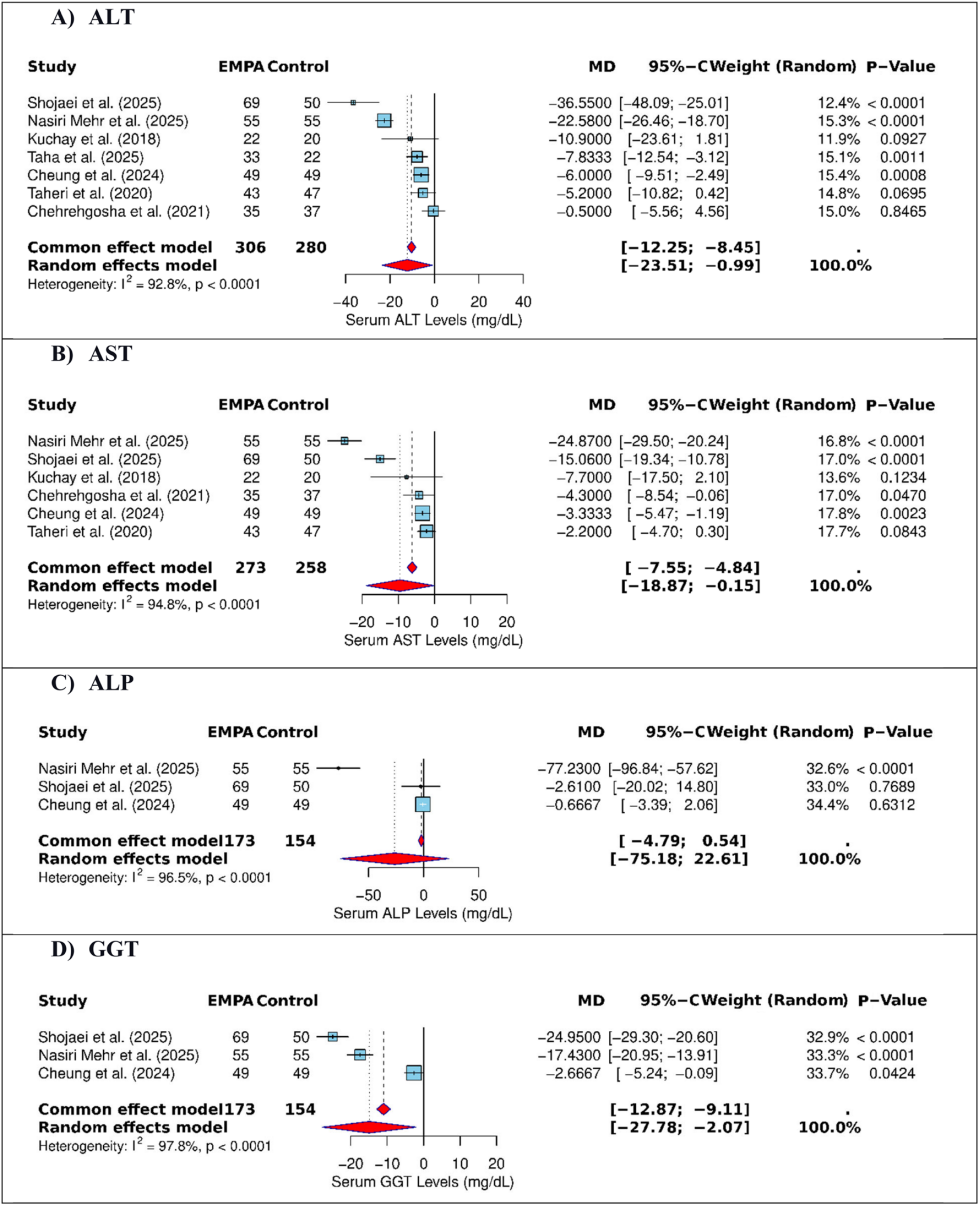
Forest plot demonstrating weighted mean difference (WMD) and 95% confidence intervals (CIs) for the impact of empagliflozin on liver enzymes (IU/L) in patients with MASLD, including: (A) aspartate aminotransferase (AST); (B) alanine aminotransferase (ALT); (C) alkaline phosphatase (ALP); (D) gamma‐glutamyl transferase (GGT).

The pooled random‐effects model analysis of seven RCTs [16, 17, 18, 25, 27, 28, 29], comprising 591 patients with MASLD, suggested that empagliflozin significantly reduced serum ALT levels (WMD: −12.24 IU/L; 95% CI: −23.51 to −0.99; *p* = 0.037), with substantial heterogeneity among studies (*I* ^2^ = 92.8%) (Figure 3A). Subgroup analyses showed that the reduction in ALT was significant in non‐diabetic patients (WMD = −6.36 IU/L, *p* = 0.014) but not in those with diabetes (*p* = 0.110), and there was no significant difference between subgroups (*p* = 0.158). ALT reduction did not significantly differ by BMI category or baseline ALT level (Table 2).

**TABLE 2:** Overall effects and subgroup meta‐analysis results for the impact of empagliflozin on serum liver enzymes, lipid profile and BMI in patients with MASLD.

	No. of studies	No. of participants	WMD (95% CI)	*p*	Heterogeneity		
					*p* heterogeneity	*I* ^2^	*p* between sub‐groups
Alanine aminotransferase (ALT) (IU/L)							
Overall effect	7	586	−12.24 (−23.51, −0.99)	0.037*	< 0.001	92.8%	—
*Subgroup analysis based on diabetic status*							
With DM	4	343	−17.33 (−41.94, 7.27)	0.110	< 0.001	95%	0.158
Without DM	3	243	−6.36 (−9.34, −3.39)	0.014*	0.75	0%	
*Subgroup analysis based on BMI status*							
Overweight	2	208	−14.27 (−30.52, 1.98)	0.085	< 0.001	97.4%	0.788
Obese	5	378	−11.52 (−23.33, 0.30)	0.056	< 0.001	87.6%	
*Subgroup analysis based on the mean serum baseline of ALT*							
< 40	4	270	−8.66 (−24.08, 6.75)	0.171	< 0.001	95.1%	0.361
> 40	3	216	−18.06 (−57.06, 20.95)	0.184	< 0.001	90.2%	
Aspartate aminotransferase (AST) (IU/L)							
Overall effect	6	531	−9.51 (−18.87, −0.15)	0.048	< 0.001	94.8%	—
*Subgroup analysis based on diabetic status*							
With DM	4	343	−13.22 (−27.89, 1.45)	0.064	< 0.001	93.0%	0.026
Without DM	2	188	−2.85 (−4.48, −1.23)	< 0.001*	0.49	0%	
*Subgroup analysis based on BMI status*							
Overweight	2	208	−13.99 (−150.81, 122.81)	0.417	< 0.001	98.5%	0.5394
Obese	4	323	−7.14 (−16.54, 2.25)	0.094	< 0.001	88.6%	
*Subgroup analysis based on the mean serum baseline AST*							
< 40	3	260	−3.03 (−4.56, −1.52)	< 0.001*	0.66	0%	0.006
> 40	3	271	−16.56 (−37.49, 4.37)	0.076	0.0007	86.1%	
Alkaline phosphatase (ALP) (IU/L)							
Overall effect	3	327	−26.28 (−75.18, 22.61)	0.292	< 0.001	96.5%	—
Gamma‐glutamyl transferase (GGT) (IU/L)							
Overall effect	3	327	−14.92 (−27.78, −2.07)	0.023*	< 0.001	97.8%	—
Triglycerides (TG) (mg/dL)							
Overall effect	5	377	−14.60 (−44.46, 15.25)	0.246	< 0.001	82.5%	—
*Subgroup analysis based on diabetic status*							
With DM	3	224	−27.07 (−61.45, 7.30)	0.122	0.049	66.9%	0.184
Without DM	2	153	−1.90 (−15.97, 12.16)	0.790	0.183	43.6%	
*Subgroup analysis based on BMI status*							
Overweight	2	208	−22.83 (−79.54, 33.87)	0.429	< 0.001	95.3%	0.618
Obese	3	169	−8.08 (−20.76, 4.59)	0.211	0.97	0%	
*Subgroup analysis based on the mean baseline TG*							
> 200	3	207	−25.44 (−56.93, 6.05)	0.113	0.001	85.6%	0.092
< 200	2	170	4.53 (−10.38, 19.46)	0.551	0.653	0%	
Total cholesterol (TC) (mg/dL)							
Overall effect	4	335	−8.07 (−24.49, 8.35)	0.335	0.004	83.5%	—
*Subgroup analysis based on diabetic status*							
With DM	2	182	−20.08 (−50.24, 10.09)	0.192	0.003	88.5%	0.157
Without DM	2	153	2.17 (−4.31, 8.66)	0.511	0.248	25%	
*Subgroup analysis based on BMI status*							
Overweight	2	208	−17.92 (−52.32, 16.48)	0.307	0.005	91.8%	0.321
Obese	2	127	0.33 (−10.35, 11.01)	0.951	0.087	65.7%	
Low‐density lipoprotein cholesterol (LDL‐C) (mg/dL)							
Overall effect	5	377	−8.45 (−33.33, 16.43)	0.329	< 0.001	88.5%	—
*Subgroup analysis based on diabetic status*							
With DM	3	224	−15.99 (−44.37, 12.38)	0.269	< 0.001	92.4%	0.219
Without DM	2	153	2.03 (−2.76, 6.83)	0.406	0.501	0%	
*Subgroup analysis based on BMI status*							
Overweight	2	208	−22.47 (−67.52, 22.58)	0.328	< 0.001	96.3%	0.340
Obese	3	169	−0.34 (−6.16, 5.48)	0.908	0.169	38.6%	
*Subgroup analysis based on the mean baseline LDL*							
> 100	3	250	−14.48 (−44.33, 15.36)	0.342	< 0.001	93.1%	0.378
< 100	2	127	−0.62 (−8.42, 7.19)	0.877	0.074	68.8%	
High‐Density Lipoprotein Cholesterol (HDL‐C) (mg/dL)							
Overall effect	5	377	2.2268 (−1.71, 6.17)	0.191	< 0.001	87.3%	—
*Subgroup analysis based on diabetic status*							
With DM	3	224	3.21 (−1.28; 7.70)	0.162	< 0.001	89.6%	0.254
Without DM	2	153	0.27 (−2.01; 2.56)	0.813	0.670	0.0%	
*Subgroup analysis based on BMI status*							
Overweight	2	208	3.6531 (−3.36, 10.66)	0.307	< 0.001	95.1%	0.442
Obese	3	169	0.7832 (−1.32; 2.89)	0.466	0.975	0.0%	
*Subgroup analysis based on mean baseline HDL*							
> 50	2	224	3.21 (−1.28, 7.70)	0.162	0.670	0.0%	0.254
< 50	3	153	0.27 (−2.01, 2.56)	0.813	< 0.001	89.6%	
Body mass index (BMI) (kg/m^2^)							
Overall effect	4	315	−0.71 (−1.00, −0.43)	< 0.001*	0.694	0.0%	—
*Subgroup analysis based on diabetic status*							
With DM	1	72	−0.91 (−2.05, 0.25)	0.124	—	—	0.743
Without DM	3	243	−0.71 (−0.97, −0.46)	< 0.001*	0.511	0.0%	
*Subgroup analysis based on BMI status*							
Overweight	1	98	−0.83 (−1.18, −0.48)	< 0.001*	—	—	0.329
Obese	3	217	−0.58 (−0.95, −0.20)	0.002*	0.694	0.0%	

*Note:* Bold values with asterisk (*): statistically significant (*p*‐value < 0.05).

Abbreviations: ALP, alkaline phosphatase; ALT, alanine aminotransferase; AST, aspartate aminotransferase; BMI, body mass index; CI, confidence interval; DM, diabetes mellitus; ES, effect size; GGT, gamma‐glutamyl transferase; HDL‐C, high‐density lipoprotein cholesterol; IU/L, international unit per litre; LDL‐C, low‐density lipoprotein cholesterol; mg/dL, milligram per deciliter; TC, total cholesterol; TG, triglycerides; WMD, weighted mean difference.

Similarly, the pooled analysis of six RCTs [16, 17, 18, 27, 28, 29] showed a significant decrease in AST levels (WMD = −9.51 IU/L, 95% CI: −18.87 to −0.15, *p* = 0.048; *I* ^2^ = 94.8%) (Figure 3B). Subgroup analysis revealed a greater, statistically significant reduction in non‐diabetic patients (WMD = −2.85 IU/L, *p* < 0.001), whereas the decrease in diabetic patients did not reach statistical significance (*p* = 0.064). The between‐group difference was statistically significant (*p* = 0.026). Patients with baseline AST < 40 IU/L also exhibited a significant reduction (WMD = −3.03 IU/L; *p* < 0.001), whereas those with higher baseline levels showed a non‐significant trend (Table 2).

For ALP, the pooled analysis of three RCTs (*n* = 327) using a random‐effects model indicated a non‐significant reduction (WMD = −26.28 IU/L; 95% CI: −75.18 to 22.61; *p* = 0.292). Considerable heterogeneity was observed across studies (*I* ^2^ = 96.5%) (Figure 3C). In contrast, GGT levels showed a significant decrease (WMD = −14.92 IU/L; 95% CI: −27.78 to −2.07; *p* = 0.023) (Figure 3D).

#### 3.4.2 Effect of Empagliflozin on Lipid Profile

**FIGURE 4:**
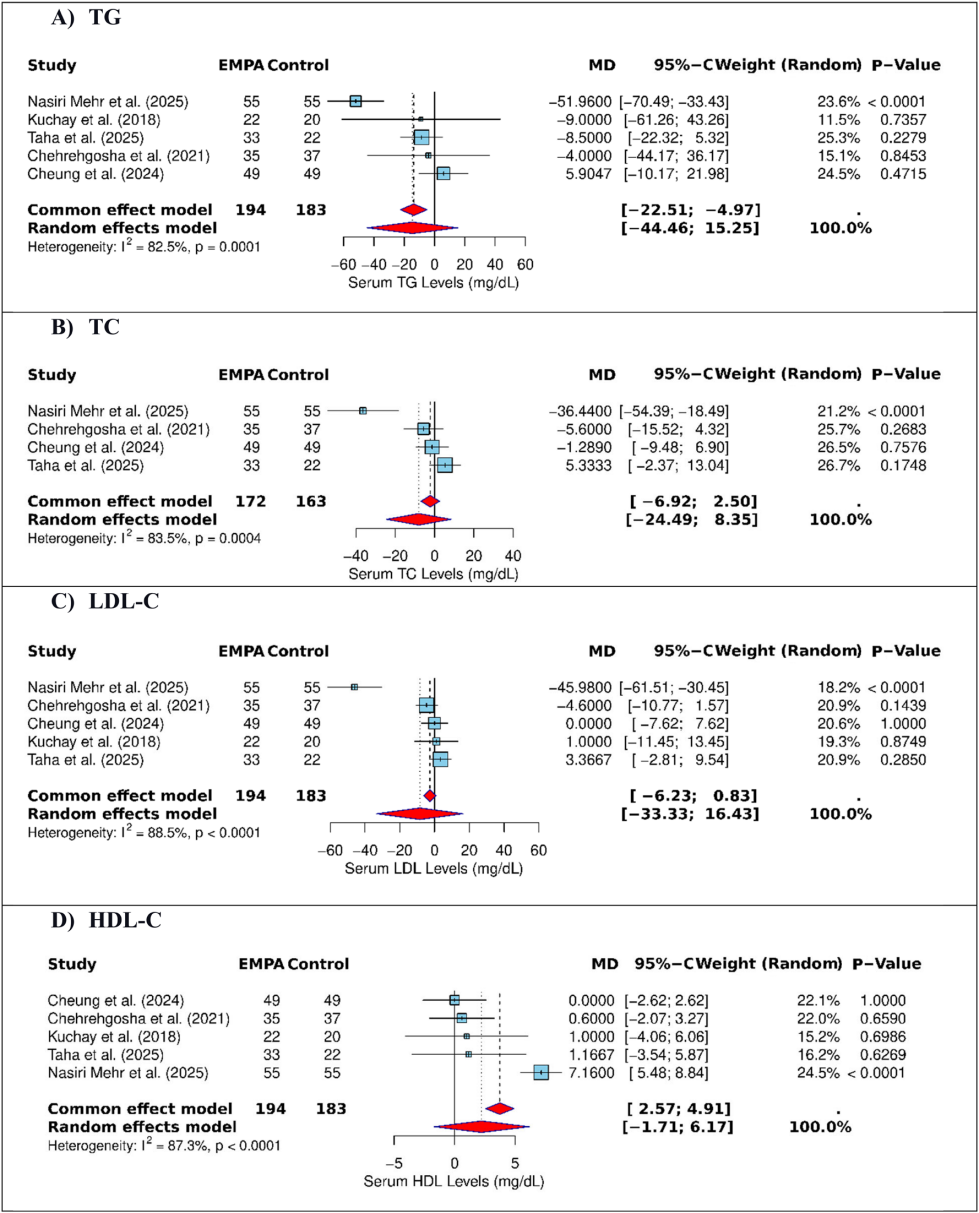
Forest plot demonstrating weighted mean difference (WMD) and 95% confidence intervals (CIs) for the impact of empagliflozin on lipid profile (mg/dL) in patients with MASLD, including: (A) (TG); (B) (TC); (C) (LDL‐C); (D) (HDL‐C).

The meta‐analysis of five RCTs comprising 377 patients with MASLD showed that empagliflozin did not significantly reduce serum TG levels (WMD = −14.60 mg/dL, 95% CI: −44.46 to 15.25, *p* = 0.246), with substantial heterogeneity across studies (*I* ^2^ = 82.5%) (Figure 4A). Subgroup analyses by diabetic status indicated a greater, though non‐significant, reduction in TG in patients with diabetes (WMD = −27.07 mg/dL, *p* = 0.122) compared with non‐diabetic patients (WMD = −1.90 mg/dL, *p* = 0.790). Stratification by BMI or baseline TG levels showed trends toward greater TG reductions in overweight patients and those with baseline TG > 200 mg/dL (WMD = −25.44 mg/dL, *p* = 0.113), but these effects were not statistically significant (Table 2).

Analysis of four RCTs including 335 patients suggested no significant effect of empagliflozin on TC (WMD = −8.07 mg/dL, 95% CI: −24.49 to 8.35, *p* = 0.335; *I* ^2^ = 83.5%) (Figure 4B). Subgroup analyses by diabetic status showed no significant reduction in patients with diabetes (WMD = −20.08 mg/dL, *p* = 0.192) and negligible change in non‐diabetic patients (WMD = 2.17 mg/dL, *p* = 0.511). Similarly, stratification by BMI did not yield significant differences, with numerically smaller reductions in overweight patients and virtually no change in obese participants (Table 2).

The pooled data from five RCTs (377 participants) indicated that empagliflozin did not significantly alter LDL‐C levels (WMD = −8.45 mg/dL, 95% CI: −33.33 to 16.43, *p* = 0.329; *I* ^2^ = 88.5%) (Figure 4C). Subgroup analyses revealed larger, non‐significant reductions in patients with diabetes (WMD = −15.99 mg/dL, *p* = 0.269) and overweight patients (WMD = −22.47 mg/dL, *p* = 0.328), whereas no meaningful change was observed in non‐diabetic or obese patients. Baseline LDL stratification (> 100 vs. < 100 mg/dL) also showed no significant differences (Table 2).

Analysis of five RCTs including 377 patients showed a non‐significant increase in HDL‐C with empagliflozin (WMD = 2.23 mg/dL, 95% CI: −1.71 to 6.17, *p* = 0.191; *I* ^2^ = 87.3%) (Figure 4D). Subgroup analyses indicated slightly greater increases in HDL‐C among patients with diabetes (WMD = 3.21 mg/dL, *p* = 0.162) and among those with baseline HDL > 50 mg/dL, but these trends did not reach statistical significance. BMI‐based subgroup analysis similarly revealed non‐significant increases in both overweight and obese participants (Table 2).

#### 3.4.3 Effect of Empagliflozin on BMI

**FIGURE 5:**
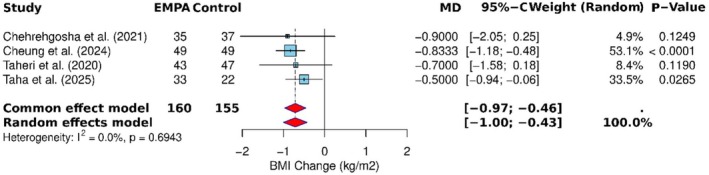
Forest plot demonstrating weighted mean difference (WMD) and 95% confidence intervals (CIs) for the impact of empagliflozin on body mass index (BMI) (kg/m^2^) in patients with MASLD.

The random‐effects analysis of four RCTs [17, 25, 28, 29] comprising 315 patients with MASLD showed that Empagliflozin significantly reduced BMI (WMD −0.71 kg/m^2^; 95% CI: −1.00 to −0.43, *p* < 0.001) and showed negligible heterogeneity (*I* ^2^ = 0%) (Figure 5). Furthermore, subgroup analyses confirmed consistent findings among patients with non‐diabetic MASLD (WMD = −0.71 kg/m^2^, *p* < 0.001), patients with overweight (WMD = −0.83 kg/m^2^, *p* < 0.001), and patients with obesity (WMD = −0.58 kg/m^2^, *p* = 0.002) (Table 2).

### 3.5 Sensitivity Analysis

**FIGURE 6:**
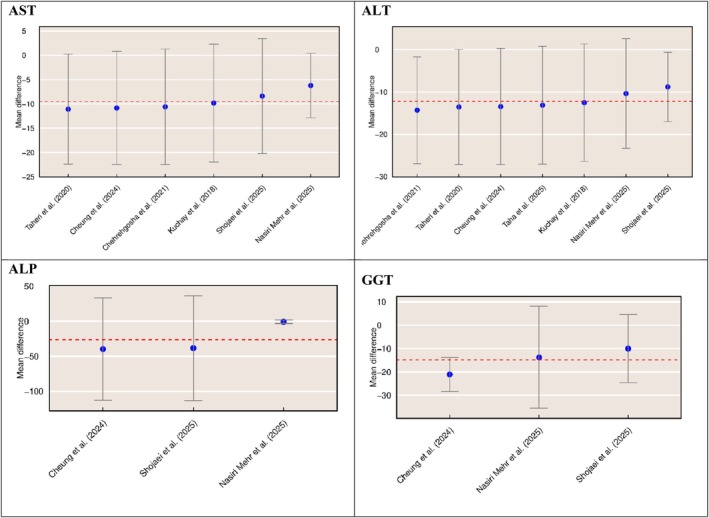
Sensitivity analysis for the effect of empagliflozin on serum lipid profile, including TG, TC, LDL‐C and HDL‐C, in patients with MASLD.

**FIGURE 7:**
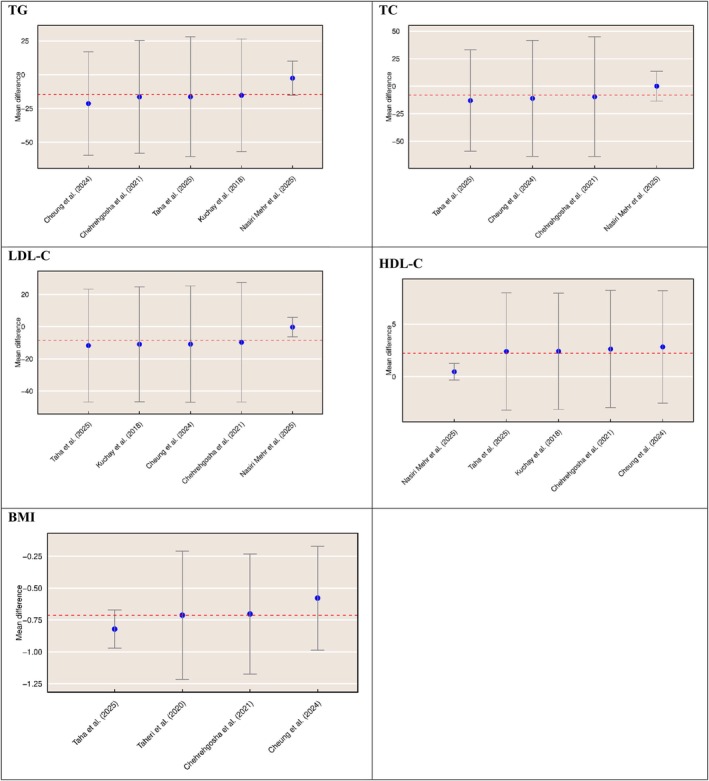
Sensitivity analysis for the effect of empagliflozin on serum lipid profile, including TG, TC, LDL‐C, HDL‐C and body mass index (BMI) (kg/m^2^) in patients with MASLD.

Based on the findings presented in Table 3, Figures 6 and 7, the sensitivity analysis indicated that the WMD results for liver enzymes (ALT, AST, ALP, and GGT), lipid profiles (TG, TC, LDL‐C and HDL‐C), and BMI were generally consistent across studies, suggesting that no single study significantly influenced the overall outcomes.

**TABLE 3:** Sensitivity analysis, trim‐and‐fill method results and publication bias (by the Begg test).

Outcome	Sensitivity analysis	Trim‐and‐fill method results	Begg's test (*p*)
ALT	Taheri et al. [28]: WMD = −13.55 (−27.14; 0.047), *p* = 0.050, *I* ^2^ = 95.4% Cheung et al. [17]: WMD = −13.46 (−27.19; 0.27), *p* = 0.053, *I* ^2^ = 94.5% Taha et al. [25]: WMD = −13.13 (−27.048; 0.79), *p* = 0.059, *I* ^2^ = 95.4% Kuchay et al. [27]: WMD = −12.53 (−26.40; 1.35), *p* = 0.068, *I* ^2^ = 96.2% Nasiri Mehr et al. [16]: WMD = −10.37 (−23.32; 2.59), *p* = 0.095, *I* ^2^ = 93.9%	2 missing studies on the left side	0.209
AST	Taheri et al. [28]: WMD = −11.09 (−22.41; 0.24), *p* = 0.053, *I* ^2^ = 94.8% Cheung et al. [17]: WMD = −10.85 (−22.50; 0.80), *p* = 0.061, *I* ^2^ = 94.7% Chehrehgosha et al. [29]: WMD = −10.59 (−22.45; 1.27), *p* = 0.068, *I* ^2^ = 96.5% Kuchay et al. [27]: WMD = −9.82 (−21.95, 2.30), *p* = 0.088, *I* ^2^ = 97.1% Shojaei et al. [18]: WMD = −8.39 (−20.18; 3.40), *p* = 0.120, *I* ^2^ = 96.5% Nasiri Mehr et al. [16]: WMD = −6.22 (−12.85; 0.41), *p* = 0.06, *I* ^2^ = 89.01%	1 missing study on the left side	0.872
ALP	Nasiri Mehr et al. [16]: WMD = −0.71 (−3.40; 1.98) *p* = 0.60, *I* ^2^ = 0%	—	0.348
GGT	Nasiri Mehr et al. [16]: WMD = −13.74 (−35.57; 8.10), *p* < 0.001, *I* ^2^ = 98.66% Shojaei et al. [18]: WMD = −10.0 (−24.46; 4.47), *p* < 0.001, *I* ^2^ = 97.72%	—	< 0.001*
TG	Nasiri Mehr et al. [16]: WMD = −2.59 (−15.2; 10.0), *p* = 0.561, *I* ^2^ = 10.87%	1 missing study on the left side	0.784
TC	Nasiri Mehr et al. [16]: WMD = 0.035 (−13.5; 13.6), *p* = 0.992, *I* ^2^ = 36.42%	1 missing study on the left side	< 0.001*
LDL‐C	Nasiri Mehr et al. [16]: WMD = −0.29 (−6.28; 5.69), *p* = 0.884, *I* ^2^ = 24.5%	1 missing study on the left side	**0.013**
HDL‐C	Nasiri Mehr et al. [16]: WMD = 0.47 (−0.31; 1.25), *p* = 0.149, *I* ^2^ = 0%	1 missing study on the left side	0.416
BMI	Taha et al. [25]: WMD = −0.82 (−0.97; −0.67), *p* = 0.002, *I* ^2^ = 0%	1 missing study on the left side	0.876

*Note:* Bold values with asterisk (*): statistically significant (*p*‐value < 0.05).

Abbreviations: ALP, alkaline phosphatase; ALT, alanine aminotransferase; AST, aspartate aminotransferase; BMI, body mass index; GGT, gamma‐glutamyl transferase; HDL‐C, high‐density lipoprotein cholesterol; LDL‐C, low‐density lipoprotein cholesterol; TC, total cholesterol; TG, triglycerides.

### 3.6 Publication Bias

**FIGURE 8:**
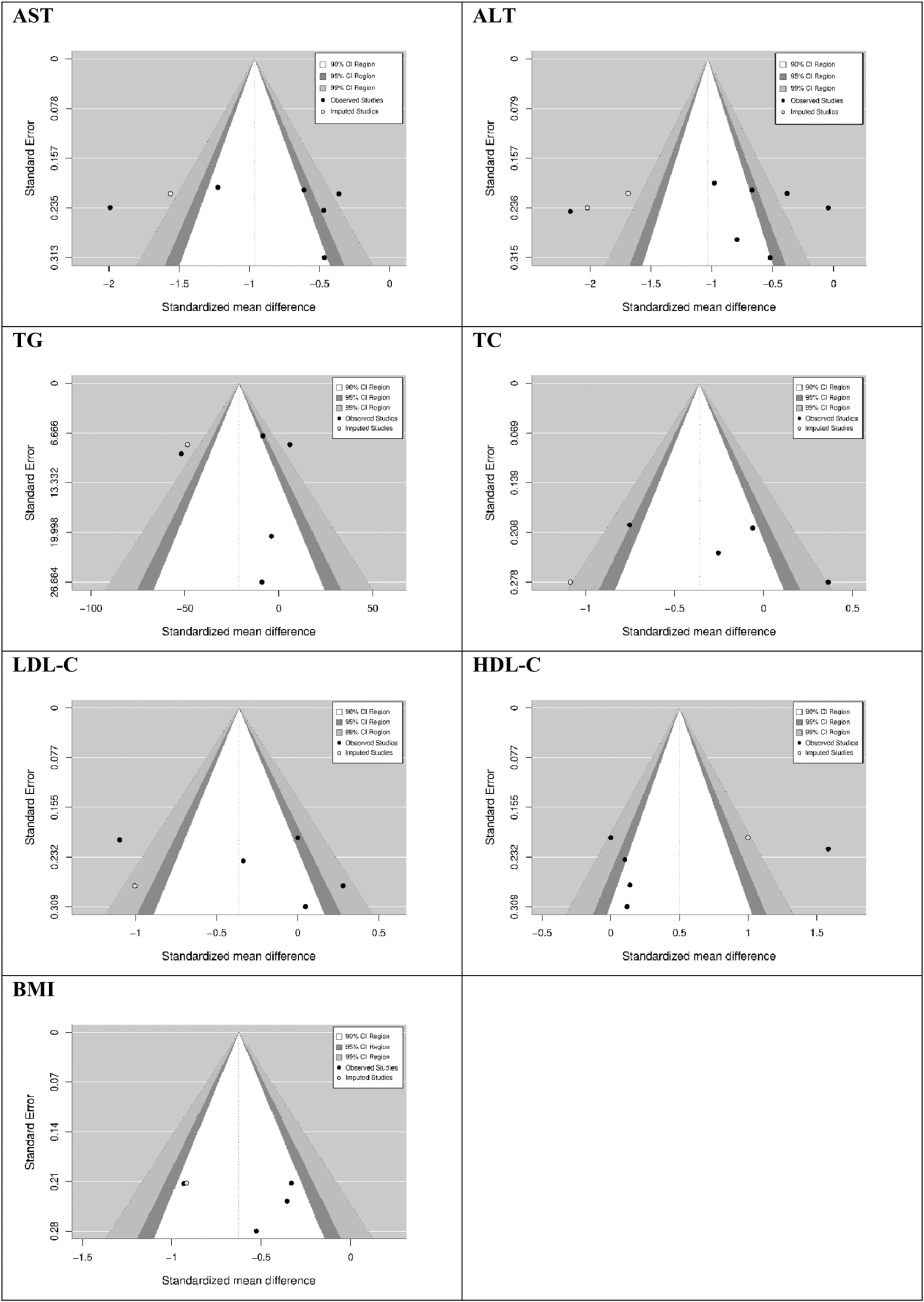
Funnel plots of publication bias for the effect of empagliflozin on AST, ALT, TG, TC, LDL‐C, HDL‐C and BMI in patients with MASLD.

The publication bias was assessed using Begg's test, and the trim‐and‐fill method is summarized in Table 3 and illustrated as funnel plots in Figure 8. For ALT, AST, TG, HDL‐C and BMI, Begg's test showed non‐significant results (*p* > 0.05), suggesting no apparent publication bias, though a few missing studies were detected on the left side of the funnel plots by the trim‐and‐fill method. Conversely, significant publication bias was observed for GGT and TC (*p* < 0.001), indicating potential asymmetry in the data. One missing study was also identified for LDL‐C (*p* = 0.013), implying a minor publication bias. Overall, although most biochemical outcomes showed no substantial publication bias, the findings for GGT, TC and LDL‐C should be interpreted with caution due to potential small‐study effects and asymmetric funnel plots.

### 3.7 GRADE Certainty Evidence

The GRADE assessment of empagliflozin in patients with MASLD revealed varying levels of certainty across outcomes (Table 4). Empagliflozin treatment was associated with moderate‐quality evidence for reductions in ALT, AST and ALP, although all showed very serious inconsistency due to high heterogeneity (*I* ^2^ > 80%). Very low‐quality evidence was observed for GGT and lipid profile (TG, TC, LDL‐C and HDL‐C), primarily due to serious imprecision and publication bias. In contrast, BMI suggested high certainty of evidence and no serious limitations across the GRADE domains.

**TABLE 4:** GRADE profile of meta‐analysis of empagliflozin in patients with MASLD/NAFLD/NASH.

Outcome	Risk of bias	Inconsistency	Indirectness	Imprecision	Publication bias	WMD (95% CI)	Quality of evidence
ALT	No serious limitation	Very serious limitation^a^	No serious limitation	No serious limitation	No serious limitation	−12.24 (−23.50, −0.99)	⊕⊕◯◯ Moderate
AST	No serious limitation	Very serious limitation^a^	No serious limitation	No serious limitation	No serious limitation	−9.51 (−18.87, −0.15)	⊕⊕◯◯ Moderate
ALP	No serious limitation	Very serious limitation^a^	No serious limitation	No serious limitation	No serious limitation	−26.28 (−75.18, 22.61)	⊕⊕◯◯ Moderate
GGT	No serious limitation	Very serious limitation^a^	No serious limitation	No serious limitation	Serious limitation^c^	−14.92 (−27.78, −2.07)	⊕◯◯◯ Very low
TG	No serious limitation	Very serious limitation^a^	No serious limitation	Serious limitation^b^	No serious limitation	−14.60 (−44.46, 15.25)	⊕◯◯◯ Very low
TC	No serious limitation	Very serious limitation^a^	No serious limitation	Serious limitation^b^	Serious limitation^c^	−8.07 (−24.49, 8.35)	⊕◯◯◯ Very low
LDL‐C	No serious limitation	Very serious limitation^a^	No serious limitation	Serious limitation^b^	No serious limitation	−8.45 (−33.33, 16.43)	⊕◯◯◯ Very low
HDL‐C	No serious limitation	Very serious limitation^a^	No serious limitation	Serious limitation^b^	No serious limitation	2.22 (−1.71, 6.17)	⊕◯◯◯ Very low
BMI	No serious limitation	No serious limitation	No serious limitation	No serious limitation	No serious limitation	−0.71 (−0.97, −0.46)	⊕⊕⊕⊕ High

Abbreviations: ALP, alkaline phosphatase; ALT, alanine aminotransferase; AST, aspartate aminotransferase; BMI, body mass index; CG, control group; CI, confidence interval; GGT, gamma‐glutamyl transferase; HDL‐C, high‐density lipoprotein cholesterol; IG, intervention group; LDL‐C, low‐density lipoprotein cholesterol; TC, total cholesterol; TG, triglycerides; WMD, weighted mean difference.

^a^ There is a high level of heterogeneity (*I* ^2^ > 80%).

^b^ There is no evidence of a significant effect of Empagliflozin treatment in MASLD.

^c^ There is a significant publication bias by Begg's test.

## 4 Discussion

### 4.1 Aim and Main Findings

To our knowledge, this is among the first updated RCT‐only meta‐analyses specifically designed to evaluate the effects of empagliflozin on routinely measured biochemical hepatic outcomes, comprehensive lipid parameters and BMI in adults with MASLD both with and without T2DM. Unlike previous reviews, which primarily focused on imaging‐derived hepatic outcomes or diabetic populations [30, 31], our analysis provides complementary evidence regarding clinically accessible biochemical markers. Pooled analyses suggested empagliflozin was associated with significant reductions in ALT, AST and GGT, findings consistent with attenuation of hepatocellular injury. Empagliflozin also significantly reduced BMI, although the magnitude of reduction was modest and likely of limited independent clinical relevance, supporting its possible role in mitigating obesity‐related metabolic stress associated with MASLD progression. Although reductions in ALP and most lipid parameters were observed, these effects were generally not statistically significant. In addition, the findings for several enzyme and lipid outcomes should be interpreted with caution because substantial heterogeneity was present across studies (*I* ^2^ frequently > 80%–90%), and publication bias was detected for GGT, TC and LDL‐C. Most included RCTs were conducted before adoption of the MASLD nomenclature and therefore used historical NAFLD or NASH terminology. Nevertheless, the enrolled populations fulfilled metabolic characteristics consistent with the current MASLD definition. Although this evolution in terminology may contribute to clinical heterogeneity, we considered the study populations sufficiently representative of contemporary MASLD and therefore did not downgrade the certainty of evidence for indirectness. Therefore, while the overall findings are generally consistent with emerging evidence suggesting that SGLT‐2 inhibitors offer metabolic and hepatic benefits beyond glycemic control, the certainty and robustness of some pooled estimates remain limited.

Subgroup findings should be interpreted with caution, as several subgroup analyses were based on a limited number of studies and participants, resulting in reduced statistical power. Moreover, these analyses were exploratory in nature and were not adjusted for multiple comparisons, increasing the possibility of chance findings. These exploratory findings suggested that non‐diabetic individuals tended to show reductions in ALT and AST compared with diabetic participants, although overlapping confidence intervals and between‐study variability limit definitive interpretation. Similarly, participants with lower baseline BMI or liver enzyme levels appeared to suggest more favourable responses, which may reflect differences in disease severity or metabolic status. For lipid outcomes, trends toward improvement in triglyceride and HDL‐C levels were observed in diabetic and overweight subgroups, although these findings were not statistically significant. Reductions in BMI were generally observed across subgroups, suggesting a potentially consistent metabolic effect of empagliflozin. Overall, these subgroup findings should be considered hypothesis‐generating rather than confirmatory, and larger well‐designed studies are needed to clarify potential subgroup‐specific effects and underlying mechanisms.

Across the included trials, limited reporting of concomitant lipid‐lowering therapy suggests minimal confounding by statin use, as no between‐group differences were observed in the two studies that reported these data (Chehrehgosha et al. [29]: baseline *p* = 0.16, final *p* = 0.780; Taheri et al. [28]: *p* = 0.869). Therefore, the overall neutral effect of empagliflozin on lipid profiles is unlikely to be substantially attributable to imbalances in background statin therapy, although incomplete reporting across studies should be considered when interpreting these findings.

### 4.2 Comparison With Previous Studies

The present meta‐analysis showed that in patients with MASLD, empagliflozin significantly reduced serum AST, ALT and GGT levels, with a nonsignificant trend toward lower ALP. Similar findings from a previous meta‐analysis showed a significant decrease in AST and liver fibrosis following empagliflozin administration. Also, although the change in ALT level did not reach statistical significance, a decreasing trend was observed among patients with NAFLD compared with the control group [19]. Another study in patients with NAFLD and diabetes concluded that empagliflozin led to biochemical improvements in the liver, significantly decreasing AST, ALT and GGT levels [32]. However, previous studies were mainly focused on diabetic patients. Maybe the reason is that the focus has shifted only recently to administering empagliflozin to patients without diabetes [17, 25, 27]. While current research included a notable number of both diabetic and non‐diabetic participants, it suggested that empagliflozin appeared to reduce hepatic enzymes and liver function more among non‐diabetic MASLD patients than among those with T2DM. Moreover, this meta‐analysis revealed a characteristic pattern in which ALT decreases more than AST, often associated with reduced liver steatosis [33]. The result is consistent with a previous meta‐analysis showing a consistent reduction in ALT than in AST with empagliflozin treatment compared to placebo [34].

This meta‐analysis found no statistically significant changes in serum lipid levels, including TG, TC, LDL‐C or HDL‐C, following empagliflozin treatment. While pooled estimates showed trends toward modest reductions in TG, TC and LDL‐C, and a slight increase in HDL‐C, these did not reach statistical significance and should be interpreted with caution. These findings are consistent with previous meta‐analyses reporting non‐significant lipid changes [19, 20]. Preclinical studies suggest that SGLT‐2 inhibitors may improve lipid metabolism and reduce lipid synthesis in NAFLD models [35], and individual trials with dapagliflozin have reported lipid‐lowering effects in patients with diabetes [36]. However, few clinical studies have rigorously evaluated these outcomes, and substantial heterogeneity in baseline lipid levels, T2DM status, BMI and concomitant lipid‐lowering therapy likely contributes to variability across studies. Further research is needed to clarify the effects of empagliflozin on lipid metabolism in MASLD.

Regarding BMI, empagliflozin produced a statistically significant reduction, although the magnitude of the decrease was not clinically meaningful. Another meta‐analysis found that empagliflozin was likely to reduce weight in patients with NAFLD compared with controls, although the magnitude of the difference was small [19]. Many studies report that treatment with empagliflozin results in a modest weight reduction [37, 38, 39]. The observed reduction, however, is suboptimal relative to the expected effect of glycosuria, suggesting the involvement of compensatory mechanisms, such as increased appetite and food intake, that mitigate further weight loss [40]. Moreover, consistent with our findings on liver enzymes, the positive effect on BMI was observed exclusively in the non‐diabetic patient group. Furthermore, the amount of BMI reduction during empagliflozin treatment was greater in overweight individuals than in those with obesity. Previous studies have suggested that dietary habits and lifestyle factors contribute to variation in the weight‐loss response to empagliflozin [41]. It is possible that the less healthy lifestyle of obese patients compared to those who are overweight may be the reason for this difference in the medication's efficacy.

Beyond SGLT2 inhibitors, several pharmacological therapies have shown promise in MASLD/NAFLD [7, 8, 9]. For example, orlistat has been reported to improve hepatic steatosis, liver enzymes, anthropometric measures and several lipid parameters, although it may increase serum triglyceride levels. In addition, emerging antisense oligonucleotides (ASOs) targeting apolipoprotein C‐III (APOC3), particularly volanesorsen, have demonstrated marked reductions in triglyceride and APOC3 levels without significantly increasing the overall risk of adverse events in patients with hypertriglyceridemia [42]. Collectively, these findings suggest that multiple pharmacological strategies targeting distinct metabolic pathways may provide complementary therapeutic benefits in patients with MASLD, highlighting the evolving landscape of disease management.

Two recent meta‐analyses have evaluated the efficacy of empagliflozin in patients with MASLD/NAFLD. Hamzah et al. [30] included 11 RCTs but restricted their analysis to patients with type 2 diabetes and primarily focused on hepatic steatosis, liver stiffness, fibrosis, glycemic control and inflammatory markers. In contrast, our study included adults with MASLD both with and without T2DM and specifically evaluated liver enzymes (including GGT and ALP), a comprehensive lipid profile (TG, TC, LDL‐C and HDL‐C) and BMI. Similarly, AlHussaini [31] analysed eight RCTs and mainly emphasized hepatic steatosis and fibrosis indices alongside selected metabolic outcomes. While both previous reviews reported improvements in some hepatic outcomes, our updated analysis demonstrated significant reductions in ALT, AST, GGT and BMI, while confirming no significant effects on serum lipid parameters. Therefore, our study complements the existing evidence by providing an updated RCT‐only synthesis with a distinct focus on routinely measured biochemical liver markers and lipid outcomes in a broader MASLD population, including both diabetic and non‐diabetic patients.

Several individual RCTs also reported improvements in direct hepatic outcomes, including reductions in liver fat quantified by magnetic resonance imaging proton density fat fraction (MRI‐PDFF), improvements in controlled attenuation parameter (CAP), and reductions in liver stiffness measured by transient elastography. Although these findings support the observed biochemical improvements, such imaging outcomes were inconsistently reported and therefore could not be quantitatively synthesized. Furthermore, no included randomized trial incorporated paired liver histology, precluding definitive conclusions regarding fibrosis regression or disease modification.

### 4.3 Underlying Mechanism

MASLD is a multifactorial condition driven by the interaction of genetic predisposition, metabolic abnormalities and environmental triggers such as overnutrition [43, 44]. This pathogenic synergy leads to hepatic fat accumulation and lipotoxicity [45]. The persistent inflammatory state ultimately drives both liver and systemic complications [46]. Therefore, it is essential to target all these connected elements in MASLD management. Empagliflozin has a significant hepatoprotective effect. Studies showed that empagliflozin reduces serum ALT levels by improving glycemia, with a significant correlation between ALT reduction and changes in fasting glucose and HbA1c [47]. Given that liver enzyme levels, especially ALT, closely reflect hepatic fat content, the observed decreases in liver enzyme levels following empagliflozin treatment align with reduced liver fat accumulation [33].

Although experimental studies have reported modest increases in LDL‐C following SGLT2 inhibitor therapy [48], our pooled analysis demonstrated no statistically significant effect on LDL‐C, suggesting that these pharmacodynamic changes were not consistently observed across clinical trials in patients with MASLD.

The medication also reduces weight gain and adiposity by increasing energy expenditure and promoting browning of adipose tissue [49]. It also induces urinary glucose excretion, thereby decreasing blood glucose and insulin levels [50, 51]. This medication improves insulin sensitivity by enhancing pancreatic β‐cell function [52, 53] and promoting catabolic pathways, such as fatty acid oxidation in skeletal muscle [54]. These effects may contribute to improved body composition by reducing caloric intake and increasing fat oxidation [49].

### 4.4 Clinical Implication

These findings support considering empagliflozin as an adjunctive treatment option in appropriately selected patients with MASLD who have approved indications for SGLT2 inhibitor therapy, particularly T2DM or elevated cardiometabolic risk. However, current evidence remains insufficient to recommend empagliflozin solely for the treatment of MASLD. The observed BMI reduction (approximately −0.7 kg/m^2^) is statistically robust (*I* ^2^ = 0%) but modest in magnitude and likely of limited standalone clinical relevance; however, it may contribute meaningfully when combined with sustained lifestyle interventions, particularly in patients with overweight or obesity. For perspective, GLP‐1 receptor agonists consistently produce substantially greater weight loss in individuals with obesity without diabetes than the modest BMI reduction observed with empagliflozin. Therefore, while empagliflozin may provide complementary hepatic and metabolic benefits, it should not be considered an alternative to GLP‐1 receptor agonists when clinically meaningful weight reduction is the primary therapeutic goal. Instead, its role is best viewed as an adjunctive therapy within a comprehensive metabolic management strategy. In contrast, no consistent beneficial effect was observed on lipid parameters, which overall appear neutral and are further influenced by potential publication bias. Therefore, empagliflozin should currently be viewed as part of a multifactorial management strategy targeting glycemic control, weight reduction and hepatic steatosis, rather than as a proven disease‐modifying therapy for MASLD or fibrosis progression. Across the included RCTs, empagliflozin was generally well tolerated, with no consistent increase in serious adverse events or treatment discontinuation compared with control groups. Overall attrition rates were low and broadly comparable between intervention and control arms. However, adverse event reporting was inconsistent across studies, preventing a formal quantitative synthesis of safety outcomes.

### 4.5 Strengths and Limitations

This study's major strength lies in its comprehensive synthesis of RCTs, employing a rigorous GRADE‐based assessment to ensure high methodological quality and confidence in the pooled outcomes. The inclusion of both diabetic and non‐diabetic MASLD populations enhances the external validity of the findings, while subgroup analyses provide valuable insights into differential therapeutic responses. Furthermore, the focus on a broad range of biochemical markers, including rarely analysed enzymes such as ALP and GGT, enhances understanding of empagliflozin's hepatic effects.

However, several limitations should be acknowledged. Considerable heterogeneity was observed for some outcomes, likely reflecting differences in T2DM status, baseline liver enzyme and lipid levels, BMI categories, diagnostic criteria for MASLD and background pharmacological and lifestyle interventions across the included trials. The relatively short intervention durations and modest sample sizes may further limit the generalizability of the findings and reduce the power to detect clinically meaningful between‐group differences, particularly for lipid outcomes. In addition, lifestyle and dietary factors were incompletely reported, and residual confounding cannot be excluded. Finally, although several individual trials reported imaging‐based hepatic outcomes such as MRI‐PDFF or transient elastography, these outcomes were insufficiently and inconsistently reported for quantitative synthesis. Consequently, the present meta‐analysis primarily reflects biochemical surrogate markers and cannot determine whether empagliflozin improves hepatic fibrosis, histological activity or long‐term clinical outcomes. Importantly, these outcomes represent surrogate biochemical markers rather than direct evidence of histological improvement.

### 4.6 Future Research Directions

Future investigations should focus on large‐scale, multicenter RCTs with extended follow‐up durations to evaluate the long‐term hepatic and metabolic outcomes of empagliflozin in MASLD. Studies incorporating histological and imaging‐based endpoints are particularly warranted to validate biochemical improvements and clarify their correlation with fibrosis regression. Further mechanistic studies exploring empagliflozin's role in hepatic lipid metabolism, inflammation and autophagy pathways could deepen our understanding of the pathophysiology. Comparative analyses with other SGLT‐2 inhibitors or combination therapies may also identify optimal treatment regimens for diverse MASLD populations.

## 5 Conclusion

This updated meta‐analysis suggests that empagliflozin may improve liver enzyme profiles and produce a statistically significant but clinically modest reduction in BMI in patients with MASLD. However, these findings are primarily based on surrogate biochemical outcomes and should be interpreted with caution given substantial heterogeneity, relatively small sample sizes, short intervention durations and evidence of publication bias for several endpoints. Larger randomized trials incorporating standardized imaging and histological outcomes are required before definitive conclusions regarding disease modification can be drawn.

## Author Contributions

**Sepehr Ramezanipour:** conceptualization, methodology, writing – original draft, funding acquisition, investigation, visualization, resources, software. **Mehdi Karimi:** conceptualization, investigation, funding acquisition, writing – original draft, methodology, software, data curation, validation, formal analysis, supervision, project administration, resources, writing – review and editing, visualization. **Pegah Abedi:** conceptualization, investigation, funding acquisition, writing – original draft, methodology, validation, resources, software, data curation. **Fereshteh Valizadeh:** resources, validation, methodology, conceptualization, investigation, funding acquisition, writing – original draft, software, data curation. **Fatemeh Naseri Rad:** conceptualization, writing – original draft, investigation, funding acquisition, software, methodology, validation, resources. **Manijeh Ebrahimzadeh Pirshahid:** data curation, resources, methodology, validation, investigation, conceptualization, funding acquisition, writing – original draft, software. **Narges Sobhan Ardekani:** software, data curation, resources, methodology, conceptualization, investigation, funding acquisition, writing – original draft, validation. **Ali Tahmasebi:** conceptualization, methodology, software, data curation, investigation, validation, formal analysis, visualization. **Meisam Gholami:** investigation, conceptualization, writing – original draft, funding acquisition, methodology, validation, resources, software, data curation.

## Funding

The authors have nothing to report.

## Ethics Statement

The authors have nothing to report.

## Consent

The authors have nothing to report.

## Conflicts of Interest

The authors declare no conflicts of interest.

## Data Availability

All data used in this meta‐analysis were extracted from published studies. The datasets supporting the findings of this study are available from the original sources cited in the manuscript. Data analysed in this meta‐analysis are included in this published article. Additional information can be provided by the corresponding author upon reasonable request.
